# Japanese evidence on Janus kinase inhibitors for rheumatoid arthritis: a narrative review of risk-optimized use

**DOI:** 10.3389/fphar.2026.1842268

**Published:** 2026-06-12

**Authors:** Haruki Matsumoto, Shuhei Yoshida, Mako Tahara, Hiroki Nibu, So Yamamoto, Takayoshi Sakamoto, Shotaro Ogawa, Kenji Saito, Yuya Sumichika, Eiji Suzuki, Tomoyuki Asano, Shuzo Sato, Yasuhiro Shimojima

**Affiliations:** 1 Department of Rheumatology and Collagen Disease, Fukushima Red Cross Hospital, Fukushima, Japan; 2 Department of Rheumatology, Fukushima Medical University School of Medicine, Fukushima, Japan; 3 Department of Rheumatology, Ohta-Nishinouchi Hospital, Fukushima, Japan

**Keywords:** herpes zoster, Janus kinase inhibitors, major adverse cardiovascular events, rheumatoid arthritis, venous thromboembolism

## Abstract

**Background:**

Janus kinase inhibitors (JAKi) have expanded treatment options for rheumatoid arthritis (RA) by providing rapid and effective oral therapy. However, their optimal use has become increasingly complex after the emergence of safety concerns involving serious infections, herpes zoster (HZ), major adverse cardiovascular events (MACE), venous thromboembolism (VTE), and malignancy. This issue is particularly relevant in Japan, where the RA population is older and has a higher prevalence of comorbidities.

**Objective:**

To review the risk-optimized use of JAKi for RA based on Japanese evidence, with particular emphasis on older patients, comorbidity-rich populations, and practical real-world treatment decision-making.

**Evidence acquisition:**

We conducted a literature search of PubMed/MEDLINE and Ichushi-Web to identify Japan-specific studies on JAKi in RA. Randomized trials, long-term extension studies, registry analyses, database studies, postmarketing surveillance reports, and observational studies were reviewed. Because of heterogeneity in design, patient background, and outcome definitions, the evidence was synthesized narratively.

**Content:**

Japanese evidence indicates that older age is an important but insufficient determinant of JAKi safety. Across studies, treatment outcomes were more strongly influenced by comorbidities, glucocorticoid exposure, laboratory abnormalities, and other patient-related risk factors. HZ emerged as the most consistent safety signal, supporting the importance of vaccination and early monitoring. By contrast, the risk of hospitalized infection was not consistently higher with JAKi than with biologic disease-modifying antirheumatic drugs in older patients, and Japanese evidence on MACE, VTE, and malignancy remained limited or inconsistent. Real-world studies also supported individualized dose optimization, whereas current data did not support routine within-class selection based primarily on JAK selectivity.

**Conclusion:**

Current Japanese evidence supports a risk-optimized approach to the use of JAKi in RA. Age alone should not determine treatment decisions. Instead, rheumatologists should individualize JAKi selection, dosing, and monitoring according to comorbidity profile, infection and vascular risk, malignancy background, and therapeutic priorities, particularly in increasingly older and multimorbid patients.

## Japanese background in the RA setting

Rheumatoid arthritis (RA) treatment has been transformed over the past two decades by the expansion of biologic disease-modifying antirheumatic drugs (bDMARDs) and Janus kinase inhibitors (JAKi), enabling tighter disease control and more proactive treat-to-target strategies. Among these options, JAKi have gained particular attention because they are administered orally and produce rapid clinical effects. In Japan, the clinical use of JAKi in RA began with the approval of tofacitinib (TOF) in 2013, followed by baricitinib (BAR) in 2017, peficitinib (PEF) in 2019, and upadacitinib (UPA) and filgotinib (FIL) in 2020 (Fukuda et al., 2022). The efficacy of JAKi has been established in several randomized controlled trials, and following these approvals, real-world studies have gradually accumulated, demonstrating a steady increase in JAKi prescribing.

This therapeutic transition has occurred within the unique demographic context of Japan, the world’s oldest society, where 29.3% of the population is aged ≥65 years and 16.8% is aged ≥75 years as of 2024 (Cabinet Office of Japan, 2025). This aging structure is directly reflected in RA care. In a nationwide analysis based on the National Database of Health Insurance Claims and Specific Health Checkups of Japan, the estimated prevalence of RA was 0.65%, and 60.8% of patients with RA were aged 65 years or older, while 7.0% were aged 85 years or older (Nakajima et al., 2020). The highest age-stratified prevalence was observed in patients aged 70–79 years (Nakajima et al., 2020). Thus, in Japan, RA management is inseparable from the realities of advanced age, multimorbidity, frailty, and concurrent safety risks. The aging of the Japanese RA population is not only a consequence of improved survival, but also reflects a shift in the age at disease onset. Using data from the National Database of Rheumatic Diseases in Japan (NinJa), Kato et al. demonstrated that the mean age at RA onset increased from 55.8 years in 2002–2003 to 57.0 years in 2007–2008 and 59.9 years in 2012–2013, with the peak age at onset shifting from the 50–59-year group to the 60–69-year group over the same period (Kato et al., 2017). More recent analyses of NinJa data showed that the use of bDMARDs and JAKi increased over time among patients with RA, and that this trend was also observed in patients with elderly-onset RA (Matsui et al., 2024).

Within this Japanese background, the role of JAKi has expanded steadily. A Japanese database study found that the total number of patients receiving JAKi increased eightfold from 2016 to 2019, with prescriptions gradually rising each year (Kaneko et al., 2023). Another claims-based study of 39,903 Japanese patients with RA treated between 2008 and 2020 likewise showed that the proportion receiving bDMARDs or JAKi increased over time after the introduction of each agent (Miyashiro et al., 2024), supporting the perception that JAKi are no longer limited salvage options but part of the current treatment strategy of RA in Japan.

**FIGURE 1:**
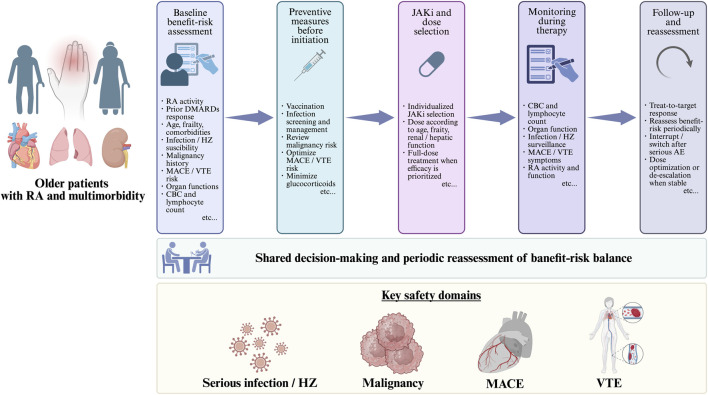
Risk-optimized use of JAKi in older patients with RA and multimorbidity. JAKi provide rapid and effective disease control in RA, but their use in older and comorbidity-rich patients requires individualized benefit-risk assessment. Baseline evaluation should include disease activity, prior treatment response, age, frailty, comorbidities, susceptibility to infection and HZ, history of malignancy, MACE/VTE risk, and relevant laboratory parameters. Before or around treatment initiation, risk-mitigation strategies, including vaccination planning, infection screening when indicated, glucocorticoid minimization, and optimization of MACE/VTE risk, should be considered. JAKi selection and dosing should be individualized according to therapeutic priorities and organ function. During treatment, laboratory monitoring, surveillance for infection and HZ, assessment of MACE/VTE symptoms, malignancy awareness, and treat-to-target evaluation should be continued. The benefit-risk balance should be reassessed longitudinally through shared decision-making. Abbreviations: CBC, complete blood count; DMARD, disease-modifying antirheumatic drug; HZ, herpes zoster; JAKi, Janus kinase inhibitors; MACE, major adverse cardiovascular events; RA, rheumatoid arthritis; VTE, venous thromboembolism. Created in BioRender. Matsumoto, H (2026) https://BioRender.com/27nw7ln.

At the same time, confidence in JAKi has been tempered by safety concerns that are especially relevant in older and comorbidity-rich populations. These concerns were brought into focus by the ORAL Surveillance trial, a post-authorization safety study in patients with RA aged 50 years or older with at least one cardiovascular risk factor, in which tofacitinib failed to demonstrate non-inferiority versus TNF inhibitors for major adverse cardiovascular events (MACE) and malignancy (Ytterberg et al., 2022). The implications are especially relevant in the Japanese context, where real-world studies have shown that older age is an independent risk factor for both MACE and malignancy in RA. Additionally, post-marketing surveillance has identified herpes zoster (HZ) and serious infections as clinically significant adverse events during JAKi therapy (Okamoto et al., 2025). Consistent with this, the systematic review underlying the 2024 Japan College of Rheumatology guideline update concluded that, although JAKi are effective in older patients with RA, safety evidence specific to this population remains limited (Sugihara et al., 2024). The current Japanese recommendations address this issue in a manner that partly parallels, but is not identical to, the European Alliance of Associations for Rheumatology (EULAR) approach (Harigai et al., 2025; Smolen et al., 2026). Whereas the EULAR recommendations state that JAKi may be considered after careful evaluation of relevant risks, the 2024 Japan College of Rheumatology guideline update incorporated separate recommendations for JAKi use and for older patients (Harigai et al., 2025; Smolen et al., 2026). Importantly, the supporting systematic review found that JAKi were effective in older patients with RA, but also highlighted the paucity of safety data specific to this population (Sugihara et al., 2024). Therefore, in Japan, JAKi use should be framed not as age-based avoidance, but as an individualized benefit-risk assessment. These considerations make Japan an especially informative setting for reconsidering how JAKi should be used in RA. The central clinical question is no longer whether JAKi are effective but rather in whom, when, and under what conditions their benefits outweigh their risks in RA treatment. In this narrative review, we therefore examine the risk-optimized use of JAKi for RA based on Japanese evidence, integrating real-world clinical considerations relevant to an increasingly older RA population. Figure 1 summarizes the risk-optimized approach proposed in this review for JAKi use in older patients with RA and multimorbidity.

## Literature search

To inform this narrative review, we conducted a structured literature search in PubMed/MEDLINE and Ichushi-Web (Japan Medical Abstracts Society) to identify Japan-specific evidence on JAKi in RA, including randomized trials, long-term extension studies, postmarketing surveillance studies, registry analyses, and observational studies. We searched for human studies published between January 2018 and March 2026, with the final search updated on 15 March 2026. Articles published in English or Japanese were considered, and the full search strategies are provided in the Supplementary File. For eligible studies for this review, we extracted information on study design, patient characteristics, baseline risk profile, comparators, exposure definition, follow-up duration, and reported outcomes. Outcomes and topics of interest included efficacy outcomes, treatment persistence, safety outcomes relevant to current risk stratification, such as serious infections, HZ, hospitalized infections, MACE, venous thromboembolism (VTE), malignancy, and laboratory abnormalities, as well as dose optimization, dose reduction, treatment sequencing, within-class JAKi selection, vaccination, comorbidity burden, renal impairment, glucocorticoid exposure, frailty-related vulnerability, and other patient-related risk factors. Because of heterogeneity in study design, patient background, outcome definitions, and analytic methods, the evidence was synthesized narratively rather than pooled quantitatively. In addition to the structured qualitative synthesis, key international trials, recent reviews, meta-analyses, and major global or regional guidance documents were selectively cited to provide a broader clinical context for the Japanese evidence base.

## Mechanistic considerations: JAK inhibition, cytokine modulation, and potential safety signals

The therapeutic rationale for JAK inhibition in RA lies in the convergence of multiple inflammatory pathways on JAK-signal transducer and activator of transcription (STAT) signaling. In RA, several cytokine systems central to synovitis and systemic inflammation signal through JAK-dependent pathways, thereby sustaining pathogenic T-cell and B-cell responses, macrophage activation, and the activated synovial tissue phenotype (Fujimoto and Niiro, 2025; Kielbowski et al., 2024). In addition, the selectivity of JAK should be interpreted cautiously. Translational studies have shown that JAKi vary in their inhibition of cytokine-induced STAT signaling, supporting the concept that relative JAK selectivity may shape at least part of the efficacy-safety balance across compounds (Taylor et al., 2024). Against this background, TOF is generally regarded as functionally JAK1/JAK3-active with some JAK2 activity, baricitinib as JAK1/JAK2-selective, upadacitinib and FIL as JAK1-preferential, and PEF as a broader pan-JAK inhibitor (Kaneko, 2020; Traves et al., 2021).

At the cytokine-profile level, JAK inhibition tends not to eliminate a single dominant mediator, but rather to compress multiple inflammatory axes simultaneously (Bonelli et al., 2024). This broader pharmacodynamic profile is clinically attractive in RA, but it also means that beneficial anti-inflammatory effects and unintended interference with host defense are mechanistically linked. Although serious infections related to immunosuppression are a general concern with DMARDs, viral infections may warrant particular attention with JAK inhibition. JAKi may increase susceptibility to viral infections by suppressing cytokine pathways involved in antiviral host defense (Adas et al., 2022). Among these, HZ has attracted particular attention. Consistent with this mechanistic rationale, long-term extension studies and integrated analyses of clinical trials of individual JAKi have consistently reported an increased incidence of HZ (Table 1) (Wollenhaupt et al., 2019; Takeuchi et al., 2021a; Taylor et al., 2022; Winthrop et al., 2020; Cohen et al., 2021; Burmester et al., 2024). In contrast, FIL has shown a numerically lower incidence of HZ than other JAKi, although direct cross-trial comparison is limited.

**TABLE 1:** Current evidence from global clinical trials, long-term extension studies, and integrated analyses on long-term safety outcomes of JAK inhibitors for RA.

Drug	Main evidence source (reference number)	Population/Exposure	Serious infection	HZ/HZ-related disease	MACE	VTE	Malignancy (excluding NMSC)
Tofacitinib	LTE/integrated safety (Wollenhaupt et al., 2019)	5 mg BID: 1,123 patients, 4,683 PY 10 mg BID: 3,358 patients, 11,608 PY	5 mg BID: 1.9/100 PY 10 mg BID: 2.6/100 PY	5 mg BID: 2.3/100 PY 10 mg BID: 3.7/100 PY	5 mg BID: 0.5/100 PY 10 mg BID: 0.4/100 PY	5 mg BID: DVT 0.1, PE 0.1/100 PY 10 mg BID: DVT 0.1, PE 0.1/100 PY	5 mg BID: 0.8/100 PY 10 mg BID: 0.8/100 PY
Peficitinib	LTE study in East Asia (Takeuchi et al., 2021a)	Max dose 50 mg/day: 36 patients Max dose 100 mg/day: 543 patients Max dose 150 mg/day: 264 patients Overall: 2,277.9 PY	50 mg/day: 4.1/100 PY 100 mg/day: 2.3/100 PY 150 mg/day: 3.0/100 PY	50 mg/day: 3.1/100 PY 100 mg/day: 6.9/100 PY 150 mg/day: 8.6/100 PY	NR	50 mg/day: 0.0/100 PY 100 mg/day: 0.1/100 PY 150 mg/day: 0.1/100 PY	50 mg/day: 1.0/100 PY 100 mg/day: 1.6/100 PY 150 mg/day: 0.6/100 PY
Baricitinib	LTE/integrated safety analyses (Taylor et al., 2022; Winthrop et al., 2020)	2 mg: 1,077 patients, 2,678 PY 4 mg: 3,401 patients, 11,872 PY	2 mg: 2.13/100 PY 4 mg: 2.62/100 PY	2 mg: 3.1/100 PY 4 mg: 4.3/100 PY	2 mg: 0.42/100 PY 4 mg: 0.54/100 PY	2 mg: DVT/PE 0.49 (DVT 0.41, PE 0.26)/100 PY 4 mg: DVT/PE 0.51 (DVT 0.35, PE 0.27)/100 PY	2 mg: 0.8/100 PY 4 mg: 1.0/100 PY
Upadacitinib	SELECT integrated analysis (Cohen et al., 2021)	15 mg: 2,630 patients, 2,655 PY 30 mg: 1,204 patients, 1,365 PY	15 mg: 3.8/100 PY 30 mg: 5.2/100 PY	15 mg: 3.7/100 PY 30 mg: 7.0/100 PY	15 mg: 0.3/100 PY 30 mg: 1.0/100 PY	15 mg: 0.6/100 PY 30 mg: 0.3/100 PY	15 mg: 0.9/100 PY 30 mg: 1.4/100 PY
Filgotinib	Integrated safety analysis (Burmester et al., 2024)	100 mg: 1,647 patients, 4,532 PY 200 mg: 2,267 patients, 8,009 PY	100 mg: 2.2/100 PY 200 mg: 1.9/100 PY	100 mg: 1.1/100 PY 200 mg: 1.5/100 PY	100 mg: 0.5/100 PY 200 mg: 0.3/100 PY	100 mg: 0.2/100 PY 200 mg: 0.2/100 PY	100 mg: 0.7/100 PY 200 mg: 0.7/100 PY

Tofacitinib groups are based on average total daily dose; peficitinib groups are categorized by maximum dose received; for baricitinib, dose-specific herpes zoster and malignancy values were supplemented from dedicated integrated analyses because the final 2022 integrated paper did not separately report those dose-specific incidence rates.Abbreviations: BID, twice daily; DVT, deep vein thrombosis; HZ, herpes zoster; LTE, long-term extension; MACE, major adverse cardiovascular events; NMSC, non-melanoma skin cancer; NR, not reported; PE, pulmonary embolism; PY, patient-years; RA, rheumatoid arthritis; VTE, venous thromboembolism.

The relationship between JAK inhibition and MACE, VTE, and malignancy is complex, and the pharmacologic basis of these associations has not yet been fully established. Similar to HZ, long-term extension studies and integrated analyses of clinical trials for individual JAKi have also reported increased incidence of serious infections, MACE, VTE, and malignancy, as summarized in Table 1. In ORAL Surveillance, TOF did not meet non-inferiority versus TNF inhibitors for MACE and malignancy in patients aged 50 years or older with at least one additional cardiovascular risk factor (Ytterberg et al., 2022). Subsequent analyses suggested that increased cardiovascular risk was particularly concentrated among patients with established atherosclerotic cardiovascular disease (Charles-Schoeman et al., 2023). ORAL Surveillance also provided important comparative data on VTE and malignancy. However, exploratory biomarker analyses did not identify a clear mechanistic explanation for the higher VTE risk observed with TOF versus TNF inhibitors, whereas malignancy analyses suggested substantial overlap between baseline cancer risk and cardiovascular risk (Charles-Schoeman et al., 2024; Curtis et al., 2023).

Taken together, these findings imply that any causal pathway is likely to reflect an interaction among patient background, comorbidity profile, and drug-specific effects rather than a single pharmacologic mechanism. This perspective may be particularly relevant in Japan, where the RA population is older and more comorbidity-rich, making individualized JAKi selection especially important in clinical practice.

## Randomized, extension, and trial-derived evidence in Japanese patients

Randomized, extension, and trial-derived studies involving Japanese patients provide important outcomes for the efficacy, dose selection, and core safety profile of JAKi in RA. The key characteristics and findings of these Japanese patient data are summarized in Table 2.

**TABLE 2:** Randomized, extension, and trial-derived evidence involving Japanese patients with rheumatoid arthritis treated with JAK inhibitors.

Drug	Main evidence source (reference number)	Population/Exposure	Serious infection	HZ/HZ-related disease	MACE	VTE	Malignancy (excluding NMSC)^*1^
Tofacitinib	Japanese LTE after phase II/III studies (Yamanaka et al., 2016)	5 mg BID: 381 patients, 1,111.7 PY 10 mg BID: 105 patients, 328.2 PY	5 mg BID: 3.2/100 PY 10 mg BID: 3.7/100 PY	5 mg BID: 7.1/100 PY 10 mg BID: 8.6/100 PY	5 mg BID: 0.4/100 PY 10 mg BID: 0.3/100 PY	NR	5 mg BID: 1.4/100 PY 10 mg BID: 0.3/100 PY
Peficitinib^*2^	LTE study in East Asia (Takeuchi et al., 2021a)	Max dose 50 mg/day: 36 patients Max dose 100 mg/day: 543 patients Max dose 150 mg/day: 264 patients Overall: 2,277.9 PY	50 mg/day: 4.1/100 PY 100 mg/day: 2.3/100 PY 150 mg/day: 3.0/100 PY	50 mg/day: 3.1/100 PY 100 mg/day: 6.9/100 PY 150 mg/day: 8.6/100 PY	NR	50 mg/day: 0.0/100 PY 100 mg/day: 0.1/100 PY 150 mg/day: 0.1/100 PY	50 mg/day: 1.0/100 PY 100 mg/day: 1.6/100 PY 150 mg/day: 0.6/100 PY
Baricitinib	Japanese subgroup analyses of phase III trials (Tanaka et al., 2018b)	RA-BEGIN: 29 patients, PY NR RA-BEAM: 93 patients, PY NR	4 mg: 1–8/100 PY	4 mg: 4–10/100 PY	4 mg: 0/100 PY	NR	4 mg: 1.0/100 PY
Upadacitinib	SELECT-SUNRISE Japanese extension study (Kameda et al., 2021)	7.5 mg: 65 patients, PY NR 15 mg: 64 patients, PY NR 30 mg: 66 patients, PY NR	7.5 mg: 4.7/100 PY 15 mg: 6.7/100 PY 30 mg: 12.7/100 PY	7.5 mg: 7.3/100 PY 15 mg: 12.3/100 PY 30 mg: 16.5/100 PY	7.5 mg: 0.8/100 PY 15 mg: 0/100 PY 30 mg: 0.9/100 PY	7.5 mg: 0/100 PY 15 mg: 0/100 PY 30 mg: 0.9/100 PY	7.5 mg: 0/100 PY 15 mg: 0.8/100 PY 30 mg: 0.9/100 PY
Filgotinib	FINCH 4 Japanese LTE analysis (Tanaka et al., 2025)	100 mg: 97 patients, 290 PY 200 mg: 110 patients, 331 PY	100 mg: 2.1/100 PY 200 mg: 2.4/100 PY	100 mg: 2.4/100 PY 200 mg: 2.7/100 PY	100 mg: 0.3/100 PY 200 mg: 0.6/100 PY	100 mg: 0/100 PY 200 mg: 0/100 PY	100 mg: 1.0/100 PY 200 mg: 0.9/100 PY

*1 In SELECT-SUNRISE, malignancy was reported as malignancy including NMSC, not as malignancy excluding NMSC.*2 Peficitinib data were derived from an analysis of patients from Japan, Korea, and Taiwan. Because Japanese patients accounted for the vast majority of the study population (95.6%), these data are discussed together with the Japanese evidence, whereas Table 1 presents global evidence. Abbreviations: BID, twice daily; HZ, herpes zoster; LTE, long-term extension; MACE, major adverse cardiovascular events; NMSC, non-melanoma skin cancer; NR, not reported; PY, patient-years; RA, rheumatoid arthritis; VTE, venous thromboembolism.

Randomized phase II studies of TOF, either in combination with MTX or as monotherapy, demonstrated clinical efficacy in Japanese patients with active RA (Tanaka et al., 2011; Tanaka et al., 2015). Subsequent long-term extension data showed sustained efficacy and a generally stable safety profile in Japanese patients treated with TOF, while also highlighting HZ as a particularly relevant safety signal in this population (Yamanaka et al., 2016). In addition, trial-derived *post hoc* analyses further suggested that TOF efficacy in Japanese patients may not be strongly affected by background MTX dose, although adverse event patterns require careful interpretation (Takeuchi et al., 2019a).

For PEF, randomized evidence from Japan and Asia is relatively extensive. A Japanese phase IIb randomized study of PEF monotherapy demonstrated dose-dependent efficacy and an acceptable short-term safety profile (Takeuchi et al., 2016). In the phase III trial, RAJ4 was conducted in Japanese patients with MTX-inadequate response, whereas RAJ3 enrolled Asian patients with inadequate response to csDMARDs, including patients from Japan (Takeuchi et al., 2019b; Tanaka et al., 2019). These trials demonstrated the efficacy of PEF 100 mg and 150 mg and supported its acceptable safety and tolerability profile, without identifying new safety signals (Takeuchi et al., 2019b; Tanaka et al., 2019). Long-term extension data from Japan, Korea, and Taiwan further supported the longer-term safety and effectiveness of PEF over a mean treatment duration of 32 months (Takeuchi et al., 2021a).

For BAR, evidence includes a Japanese phase IIb randomized, placebo-controlled study in patients receiving background MTX, along with its subsequent 64 weeks extension (Tanaka et al., 2016; Tanaka et al., 2018a). The phase IIb study demonstrated rapid improvements in signs, symptoms, and physical function in Japanese patients, with a generally acceptable safety profile and no major safety differences compared with non-Japanese populations (Tanaka et al., 2016). The extension study supported the maintenance of efficacy and safety over the longer term (Tanaka et al., 2018a). In addition, Japanese subgroup analyses of RA-BEGIN, RA-BEAM, RA-BUILD, and RA-BEACON suggested that the efficacy and overall safety profiles of BAR in Japanese patients were broadly consistent with those observed in the overall trial populations, although HZ occurred more frequently in the Japanese subpopulations of RA-BEGIN and RA-BEAM (Tanaka et al., 2018b).

The SELECT-SUNRISE study provided Japan-specific randomized dose-ranging evidence for UPA in csDMARD-inadequate responders, demonstrating efficacy and supporting the 15 mg dose as having the most favorable benefit-risk profile (Kameda et al., 2020). The 84 weeks extension further supported sustained efficacy and tolerability, with lower rates of adverse events, infections, opportunistic infections, serious infections, and HZ observed with 7.5 mg and 15 mg compared with 30 mg (Kameda et al., 2021).

For FIL, Japanese subgroup analyses from the FINCH 1, FINCH 2, and FINCH 3 phase III trials provide trial-derived evidence across clinically distinct RA populations, including MTX-inadequate responders, bDMARD-inadequate responders, and patients with limited or no prior MTX exposure (Tanaka et al., 2022; Takeuchi et al., 2022; Atsumi et al., 2022). These analyses suggested that FIL 200 mg and 100 mg were generally effective and well tolerated in Japanese patients (Tanaka et al., 2022; Takeuchi et al., 2022; Atsumi et al., 2022). Subsequent FINCH 4 long-term extension analyses further supported the durability of efficacy and the absence of unexpected new safety signals during long-term treatment in Japanese patients (Tanaka et al., 2023; Tanaka et al., 2025).

These randomized, extension, and trial-derived data are informative. However, because trial populations are selected and event numbers for uncommon outcomes are limited, these data should be interpreted alongside postmarketing surveillance, registry analyses, and observational studies when discussing risk stratification in routine Japanese practice, particularly in older patients and those with comorbidities. Compared with global trial datasets, these Japan-focused analyses are also limited by smaller sample sizes and variability in patient backgrounds, which should be considered when comparing outcomes across studies.

## Japan-specific large cohort evidence

Safety evidence from Japan-specific cohorts on JAKi has accumulated since the approval of JAKi in Japan. Among them, the most informative datasets for older patients appear to be the FIRST registry, the Kansai Consortium for Wellbeing of Rheumatic Disease Patients (ANSWER) cohort, the Kyoto University Rheumatoid Arthritis Management Alliance (KURAMA) cohort, the KEIO-RA cohort, and the Tsurumai Biologics Communication Registry (TBCR), as well as the NinJa Database, a nationwide multicenter observational database and one of the largest RA registries in Japan. The key characteristics of these cohorts are summarized in Table 3.

**TABLE 3:** Japanese cohorts and registries included in this review to inform JAK inhibitors use in RA.

Registry/Cohort	Design/Scale	Patient profile	JAKi-related contribution	Representative publications in this review
NinJa Database	Nationwide multicenter annual RA database	Broad Japanese RA population; mean age approximately mid-60s	Captures advanced-therapy exposure including bDMARDs/JAKi	Saeki et al., 2012; Matsui et al., 2025
FIRST registry	Large multicenter cohort of bDMARD/JAKi-treated RA patients	Aging and increasingly comorbidity-rich population	Provides age-stratified retention, JAKi cycling, and comparative effectiveness data	Sonomoto and Tanaka, 2025; Sonomoto et al., 2025; Kubo et al., 2023; Miyazaki et al., 2025a; Miyazaki et al., 2025b; Sonomoto et al., 2026
ANSWER cohort	Multicenter real-world registry from the Kansai region	Middle-aged to older routine-care RA population	Includes BAR/TOF retention, four-JAKi comparison, and elderly-onset RA analyses	Ebina et al., 2020; Jinno et al., 2025; Ebina et al., 2022; Hayashi et al., 2024; Ebina et al., 2023
KURAMA cohort	Single-center longitudinal cohort from Kyoto University	Aging RA population with long-term treatment-era data	Describes increasing bDMARD/JAKi use and secular trends	Fujii et al., 2024
KEIO-RA cohort	Single-center specialist-practice cohort from Keio University	Older and comorbidity-enriched RA population	Provides JAKi retention, MTX-combination, dosing, and ILD subgroup data	Takanashi et al., 2025; Suzuki et al., 2026
TBCR	Multicenter registry originally focused on biologic therapy	Older, treatment-experienced RA patients	Published JAKi data mainly focused on BAR	Kojima et al., 2012; Takahashi et al., 2020; Nagoya University Graduate School of Medicine, 2026

Only representative features relevant to JAKi use are shown. Detailed study-specific sample sizes, follow-up durations, and individual publications are described in the main text and references.

Abbreviations: BAR, baricitinib; bDMARDs, biologic disease-modifying antirheumatic drugs; ILD, interstitial lung disease; JAKi, Janus kinase inhibitors; MTX, methotrexate; RA, rheumatoid arthritis; TOF, tofacitinib.

The NinJa Database is a nationwide observational resource for RA epidemiology and safety research in Japan (Matsui et al., 2007; Saeki et al., 2012). As a nationwide, multicenter, longitudinal database with annual data collection, it is well-suited not only to evaluating safety outcomes, including malignancy, but also to assessing treatment trends, age-related changes in disease presentation, disease activity, and real-world outcomes across Japanese clinical practice (Kato et al., 2017; Saeki et al., 2012; Matsui et al., 2025). In the NinJa-based malignancy analysis included in the literature set, 26,607 patients with RA were evaluated (mean age 64.16 years), among whom 1,018 malignancies were identified (Matsui et al., 2025). Importantly, the study also included a bDMARDs- or JAKi-exposed sub-cohort, allowing treatment-stratified assessment of malignancy incidence in patients receiving advanced therapies. Across sub-cohorts, the incidence of overall malignancy remained comparable to that of the general population, with the exception of lymphoma and skin cancer (Matsui et al., 2025). Although NinJa currently offers less detailed event-level JAKi safety data beyond malignancy than other large RA cohorts, it remains a highly valuable resource for population-level risk estimation and for situating JAKi safety within real-world Japanese RA treatment practice.

The FIRST registry is a large Japanese multicenter cohort of patients with RA treated with bDMARDs or JAKi and has provided one of the most detailed longitudinal views of treatment evolution in an aging society (Sonomoto and Tanaka, 2025). Registry-wide analyses have followed approximately 5,500 patients over 20 years, and a recent 5-year analysis included 5,130 treatment courses over 16,616 person-years (Sonomoto and Tanaka, 2025; Sonomoto et al., 2025). Over time, the treated population became progressively older and more comorbidity-enriched, with mean age increasing from 51.9 to 64.3 years and the prevalence of lung disease (from 11.1% to 36.2%), prior malignancy (from 2.2% to 13.1%), MACE (from 2.2% to 9.4%), and thrombosis (from 2.2% to 4.0%) also increased (Sonomoto et al., 2025). Despite this shift, the use of bDMARDs or JAKi expanded to patients with lower disease activity, accompanied by a decrease in discontinuations due to adverse events and a decline in infection-related discontinuations from 2.1 to 0.7 per 100 person-years (Sonomoto et al., 2025). In JAKi-focused analyses from the FIRST registry, JAKi showed among the highest 3-year retention rates in patients aged <65 years and 65–74 years, and inadequate efficacy was a major reason for discontinuation across age groups (Kubo et al., 2023a). However, infection-related discontinuation increased with age and was particularly notable in patients aged ≥75 years receiving JAKi, whereas no clear increase in malignancy or MACE was observed within the registry (Kubo et al., 2023a). In a subsequent analysis of patients with inadequate response to an initial JAKi, 31.8% met criteria for JAKi inadequate response, and suboptimal JAKi dosing emerged as one contributing factor (Miyazaki et al., 2025a). Among these patients, cycling to another JAKi was associated with higher remission rates than switching to bDMARDs, with similar retention and adverse-event profiles at 26 weeks (Miyazaki et al., 2025a). A propensity-weighted analysis comparing JAKi with IL-6 receptor inhibitors also found a higher incidence of infection in the JAKi group at 26 weeks, despite similar remission rates after failure of a single prior bDMARD (Miyazaki et al., 2025b). However, better outcomes with JAKi were observed in more treatment-refractory patients (Miyazaki et al., 2025b). More recently, a 2-year FIRST analysis of 607 JAKi treatment courses reported an overall retention rate of 78%, identified insufficient effectiveness and adverse events as the leading reasons for discontinuation, and found no clinically meaningful differences in adjusted effectiveness among TOF, BAR, UPA, and FIL (Sonomoto et al., 2026).

The ANSWER cohort is a Japanese multicenter real-world registry. This cohort has been informative for treatment sequencing. After failure of first-line tocilizumab or abatacept, retention patterns of secondary bDMARDs or JAKi varied by the preceding agent, suggesting that prior bDMARDs exposure may meaningfully influence subsequent treatment durability (Ebina et al., 2020). Additionally, this cohort has served as a valuable resource for evaluating treatment persistence across bDMARDs or JAKi, as well as for examining outcomes in clinically relevant subgroups, such as elderly-onset RA (Jinno et al., 2025). In elderly-onset RA patients, IL-6 receptor inhibitors and JAKi were associated with longer retention and fewer discontinuations due to ineffectiveness than the TNF inhibitors (Jinno et al., 2025). This cohort has also provided JAKi-specific information on safety and treatment persistence. A representative JAK-focused analysis from this cohort evaluated 351 treatment courses of BAR or TOF, with a mean patient age of 60.5 years, underscoring its relevance to the middle-aged and older RA population commonly encountered in Japanese practice (Ebina et al., 2022). In a dedicated analysis of BAR and TOF, adjusted retention due to lack of effectiveness was 84.6% for BAR and 75.9% for TOF, while retention due to toxic adverse events was 82.7% and 87.5%, respectively, with no significant between-drug differences (Ebina et al., 2022). By contrast, retention due to toxic adverse events fell to 69.3% in very old patients aged ≥75 years (Ebina et al., 2022). In multivariable analysis, increasing age, female sex, and concomitant prednisolone ≥5 mg/day were associated with discontinuation due to toxic adverse events (Ebina et al., 2022). In another comparative analysis of this cohort, no significant between-drug differences were observed among TOF, BAR, PEF, and UPA in adjusted short-term retention, CDAI improvement, remission, or low disease activity, supporting the view that apparent differences among JAKi in routine practice may largely reflect case mix background rather than major efficacy differences (Hayashi et al., 2024). In a broader ANSWER analysis of 6,666 bDMARDs or JAKi treatment courses, baseline oral glucocorticoid use was again associated with toxic adverse-event-related discontinuation in JAKi-treated patients (Ebina et al., 2023).

The KURAMA cohort is a well-established single-center observational cohort (Fujii et al., 2024a). It is particularly useful for examining secular changes in RA management and outcomes across the bDMARDs or JAKi era (Fujii et al., 2024a). By 2022, the cohort had enrolled 4,418 patients, and a recent 10-year analysis included 5,070 annual cross-sectional observations and 1,816 bDMARDs or JAKi initiation episodes (Fujii et al., 2024a). The mean age of patients in the annual survey increased from 62.9 years in 2012 to 65.9 years in 2021 (Fujii et al., 2024a), again emphasizing the importance of this cohort for understanding RA care in older adults. Over the same period, glucocorticoid use decreased from 40.5% to 18.6%, whereas bDMARDs or JAKi use increased from 29.5% to 53.2%, accompanied by progressive improvement in disease activity and function (Fujii et al., 2024a). Within the KURAMA cohort, the main JAK-related finding was that JAKi use progressively increased in routine practice, whereas, among switched patients, their effectiveness was broadly comparable to that of bDMARDs, with no clear advantage in adjusted clinical or functional outcomes (Fujii et al., 2024a).

The KEIO-RA cohort is a single-center longitudinal clinical cohort from Keio University that provides detailed real-world data from routine specialist practice (Takanashi et al., 2025; Suzuki et al., 2026). Although it is a single-center study, its continuous longitudinal accumulation of cases has generated valuable and clinically relevant evidence. In JAKi-focused analyses, a general retention study evaluated 294 treatment courses in 201 patients (mean age 65.1 years) and showed a mean drug continuation duration of 17.2 ± 17.4 months (Takanashi et al., 2025). Full-dose JAKi use and concomitant methotrexate (MTX) were both associated with higher retention, while full-dose JAKi plus MTX was linked to fewer discontinuations due to lack of efficacy without an apparent increase in safety-related events (Takanashi et al., 2025). Among patients receiving full-dose JAKi with MTX, UPA and FIL had lower discontinuation rates due to lack of efficacy, whereas BAR had fewer safety-related discontinuations (Takanashi et al., 2025). JAKi safety did not show a clear age-dependent deterioration in discontinuation-based analyses (Takanashi et al., 2025). However, this study primarily evaluated safety based on treatment discontinuation and did not provide robust age-stratified incidence estimates for MACE, VTE, HZ, or incident malignancy. In the RA-associated interstitial lung disease (ILD) subgroup from this cohort, which comprised an even older population, adverse events were also broadly comparable regardless of MTX use, despite the older age of the non-MTX group (median age 78 years vs. 67 years in the MTX group; overall median age 75 years) (Suzuki et al., 2026). This analysis included 86 treatment courses, with an overall 24-month retention rate of 42.3%, and identified prior exposure to multiple bDMARDs or JAKi emerged as an independent predictor of discontinuation (Suzuki et al., 2026). Event-level data in this RA-ILD analysis showed malignancy in 2.3%, cardiovascular events in 2.3%, deep vein thrombosis in 0%, varicella-zoster virus infection in 7.0%, infection requiring hospitalization in 5.8%, and no treatment-related deaths (Suzuki et al., 2026).

The TBCR is a Japanese multicenter registry established in 2008 to evaluate long-term outcomes in patients initiating bDMARDs and has since provided real-world data on JAKi (Kojima et al., 2012; Takahashi et al., 2020). It includes over 4,000 patients with RA and represents one of the largest longitudinal registries in Japan (Nagoya University Graduate School of Medicine, 2026). A representative BAR study from this registry analyzed 113 patients with a mean age of 66.1 years and found that the 24 w discontinuation rate due to adverse events was 6.5%, highlighting its relevance to older, treatment-experienced patients (Takahashi et al., 2020). HZ developed in 7 patients, corresponding to an incidence rate of 8.4 per 100 patient-years; no severe HZ was observed (Takahashi et al., 2020). However, published TBCR analyses have so far provided limited event-level incidence data on major adverse cardiovascular events or venous thromboembolism, and reporting of malignancy has remained limited to isolated events rather than systematic, event-specific analyses.

Taken together, these cohorts indicate that a major strength of the Japanese evidence base is its unusually rich representation of older adults, making older RA not a peripheral subgroup but a central context for interpreting JAKi use in Japan. Beyond these major Japanese cohorts, several real-world Japan-based postmarketing surveillance studies and single-center and multicenter analyses have focused more directly on JAKi safety, treatment persistence, dose optimization, and use in older and clinically vulnerable subgroups with substantial comorbidity concerns.

## Nationwide postmarketing surveillance in Japan

Particularly informative are the nationwide postmarketing surveillance datasets from Japan. In a 3-year all-case surveillance study of TOF, the TOF group in the adherent comparative safety analysis set was older than the non-tofacitinib control cohort (mean age 61.1 vs. 56.8 years), serious infections were more frequent than in a non-tofacitinib control cohort (6.86 vs. 1.42 per 100 patient-years), malignancies were numerically higher (1.40 vs. 0.88 per 100 patient-years), and mortality was also higher (0.89 vs. 0.26 per 100 patient-years) (Kuwana et al., 2025). HZ was also common in the overall TOF safety analysis set, occurring in 4.73 per 100 patient-years, although a direct comparison with the non-tofacitinib control cohort was not reported (Kuwana et al., 2025). A subsequent *post hoc* analysis of the same 3-year Japanese TOF all-case surveillance, including 7,021 patients stratified by age and cardiovascular risk factors, further showed that older age was a consistent risk factor for MACE, malignancies, and serious infections, whereas MACE and serious infection risk were additionally amplified by the presence of cardiovascular risk factors (Yamaoka et al., 2026). In that analysis, the highest incidence rates were observed in patients aged ≥50 years with ≥1 cardiovascular risk factor, with incidence rates per 100 patient-years of 0.76 for MACE, 2.18 for malignancy, and 5.88 for serious infection (Yamaoka et al., 2026). Smoking history and diabetes were important contributors to MACE and malignancy risk, while hypertension, diabetes, coronary artery disease, and extra-articular RA were also associated with serious infection risk (Yamaoka et al., 2026). Likewise, in a 3-year all-case surveillance study of BAR in 4,720 Japanese patients with RA (mean age 63.9), 2,580 (54.7%) were ≥65 years old, the 3-year persistence rate was 45.4%, and incidence rates per 100 patient-years for HZ, serious infection, malignancy, MACE, and VTE were 4.68, 3.05, 1.09, 0.35, and 0.25, respectively, without evidence of increasing risk with longer exposure (Okamoto et al., 2025). For PEF, Japanese all-case surveillance is ongoing, and interim manufacturer-linked summaries indicate a large safety analysis set of 3,466 patients with a mean age of 68.9 years, again underscoring the relevance of these datasets to older Japanese RA populations seen in daily practice (Astellas Medical Net). For UPA, a 24 w interim analysis of Japanese all-case surveillance included 2,934 enrolled patients, of whom 2,732 were in the safety analysis set. Mean ages were 64.7 years in the 15 mg cohort and 68.4 years in the 7.5 mg cohort (Fujii et al., 2024b). Adverse events occurred in 21.7% and serious adverse events in 5.1%, while no new safety signals were identified (Fujii et al., 2024b). For FIL, Japan-specific postmarketing surveillance is also ongoing, and the current Japanese interview form describes a nationwide all-case surveillance program with a planned safety analysis set of 1,000 patients (Gilead Sciences, 2026).

Collectively, these nationwide surveillance data, including ongoing all-case post-marketing surveillance programs, complement registry-based cohorts by providing broader Japan-specific estimates for relatively infrequent but clinically important outcomes, while not indicating any consistent signal of worsening or newly emerging adverse events unique to the Japanese population.

## Age-related tolerability and safety considerations in Japanese RA patients treated with JAKi

Single-center, multicenter, and database-based Japanese studies have provided important insights into the age-related tolerability and broader safety profile of JAKi in Japanese patients with RA.

Our department conducted a multicenter study of 133 patients aged ≥65 years and found that overall JAKi retention and adverse-event-related discontinuation were comparable between patients aged 65–74 years and those aged ≥75 years (Temmoku et al., 2023a). In a related analysis from the same cohort, early therapy-induced lymphopenia within the first 4 months after JAKi initiation was associated with reduced drug retention due to adverse events in TOF-treated patients, suggesting that early hematologic monitoring may be particularly informative in vulnerable older adults (Temmoku et al., 2023b).

Japanese real-world studies suggest that JAKi may increase the risk of infections, particularly HZ, although this does not necessarily lead to a higher risk of hospitalization than with bDMARDs. In a nationwide claims-based study, Takabayashi et al. showed that TOF was associated with a higher incidence of HZ than bDMARDs, whereas the incidence of other opportunistic infections was broadly comparable between the groups (Takabayashi et al., 2023). Consistent with this, Uchida et al. reported that the incidence of HZ was significantly higher in JAKi-treated patients than in TNF inhibitor-treated patients, whereas the rates of other serious infectious diseases did not differ significantly between the groups (Uchida et al., 2023). In contrast, our department found that JAKi-treated patients had a higher incidence of serious infections other than HZ than IL-6 receptor inhibitor-treated patients (Yoshida et al., 2024). Beyond serious infections, a single-center Japanese real-world study by Muramatsu et al. suggested that JAKi are a risk factor for HZ and that concomitant MTX may further modify this risk (Muramatsu et al., 2023). More recently, Tabata et al. reported that recombinant zoster vaccination tended to reduce HZ incidence among Japanese RA patients receiving JAKi, with an IPTW-estimated vaccine effectiveness of 59.1%, although the reduction was not statistically significant (Tabata et al., 2025). Notably, breakthrough HZ after vaccination was associated with lower lymphocyte counts and higher disease activity (Tabata et al., 2025). This preventive perspective is complemented by the PCV13 study of Mori et al., which showed that JAKi monotherapy preserved pneumococcal vaccine immunogenicity, whereas combined MTX and JAKi attenuated antibody responses, highlighting the practical importance of vaccination timing and concomitant therapy management in older or infection-prone patients (Mori et al., 2023). Regarding hospitalized infections, an interesting analysis has been reported. Sakai et al. showed that although the absolute incidence of hospitalized infection increased markedly with age, bDMARDs or JAKi were not associated with a higher adjusted risk of hospitalized infection than MTX in older patient (Sakai et al., 2022). Likewise, Harigai et al. demonstrated that in patients aged ≥75 years, hospitalized infection rates increase substantially with age overall, but comparative risks across bDMARDs or JAKi were broadly similar, suggesting that advanced age primarily amplifies baseline susceptibility to infection rather than defining a uniquely JAKi-specific infection signal (Harigai et al., 2024).

Japanese data on MACE and VTE in JAKi-treated RA are more limited than those on infection risk and are less well stratified by age. In a large retrospective population-based study using a Japanese health insurance database, Sakai et al. reported that JAKi use was associated with a significantly higher adjusted risk of overall cardiovascular events than TNF inhibitors use (Sakai et al., 2024). By contrast, in a smaller Japanese multicenter cohort study of 499 patients treated with TOF, BAR, or TNF inhibitors, Uchida et al. found that the incidence of HZ was significantly higher in the JAKi group, whereas no significant between-group differences were observed for other adverse events, including MACE (Uchida et al., 2023). Over 959.7 patient-years of follow-up, only two MACEs were identified in JAKi-treated patients, suggesting that Japanese clinic-based cohort data have not consistently reproduced a clear excess cardiovascular signal, at least within the limits of modest sample size and event numbers (Uchida et al., 2023).

Evidence specific to VTE from Japanese real-world practice remains limited. A Japan-based case presentation and literature review by Mori et al. highlighted that VTE risk under JAKi therapy should be understood in the context of cumulative host risk factors, including advanced age, obesity, diabetes, hypertension, hyperlipidemia, smoking, comorbidities, and uncontrolled RA inflammation, rather than solely attributed to drug exposure (Mori et al., 2021). Consistent with this clinically risk-based assessment, Yoshimura et al. reported that in patients with chronic kidney disease receiving first-line JAKi, HZ and deep vein thrombosis tended to be more frequent in those with eGFR <30 mL/min/1.73 m^2^, although the increase was not statistically significant (Yoshimura et al., 2025). Taken together, the currently available Japanese evidence suggests that vascular safety assessment should be individualized, particularly in older or multimorbid patients.

Evidence regarding malignancy in Japanese patients receiving JAKi also remains limited. In the previously mentioned NinJa database analysis, the overall incidence of malignancy in Japanese patients with RA was not elevated relative to that in the general population, although lymphoma and skin cancer occurred more frequently (Matsui et al., 2025). The previously mentioned multicenter cohort study compared Japanese patients treated with TOF or BAR with those treated with TNF inhibitors, and identified 11 malignancies among JAKi-treated patients (Uchida et al., 2023). Although the overall malignancy risk in the JAKi group was higher than in the general population, the increase was not statistically significant, and no significant difference was observed between the JAKi and TNF inhibitors groups (Uchida et al., 2023).

In Japan, direct evidence linking frailty-related vulnerability to JAKi adoption remains limited. Nevertheless, a preliminary survey of Japanese rheumatologists managing late-onset RA suggests that frailty-related domains may indirectly influence treatment selection in routine practice (Takanashi et al., 2024). The survey showed that renal, cognitive, physical, and pulmonary impairment; life circumstances; comorbidities; malignancy; infection; and economic factors were considered in therapeutic decision-making, and that bDMARDs were generally preferred over JAKi due to concerns about drug metabolism, HZ, malignancy, and MACE (Takanashi et al., 2024). Notably, only 5 of 65 rheumatologists (8%) assessed frailty using validated criteria (Takanashi et al., 2024), indicating that frailty may be clinically recognized but not systematically measured in Japanese RA practice. Thus, the relationship between frailty-related vulnerability and JAKi adoption remains an important topic for future investigation in Japanese real-world practice.

Collectively, these studies suggest that age alone does not fully explain treatment outcomes. Rather, tolerability appears to be influenced by a broader range of factors, including comorbidity burden, laboratory abnormalities, and other host-related vulnerabilities. Regarding infection, preventive strategies such as vaccination with vaccines approved in Japan, including the HZ vaccine, are likely to play an important role.

## Dose optimization and de-escalation of JAKi in Japanese routine practice

Japanese real-world studies also support an approach to JAKi dose optimization that encompasses both dose reduction in older patients and maintenance of full-dose therapy when effectiveness is the primary concern. In the older cohort reported by our department, overall retention was significantly lower among patients treated at the approved dose group than in the reduced-dose or tapered-dose group, suggesting that de-intensification may improve treatment persistence in selected older patients with greater vulnerability to adverse events (Temmoku et al., 2023a). Renal function is another recurring determinant of individualized dosing. Maeyama et al. showed that BAR 2 mg in patients with moderate renal impairment achieved efficacy, retention, and safety comparable to those of BAR 4 mg in patients with normal or mildly impaired renal function (Maeyama et al., 2024). Similarly, Tanno et al. reported sustained effectiveness and acceptable safety of UPA across clinically important subgroups, including patients aged ≥75 years and those with renal impairment, with 7.5 mg/day more frequently selected in these groups, supporting the feasibility of adjusted dose selection in routine practice (Tanno et al., 2026).

Dose optimization after response has also been prospectively examined. Mori and Ueki showed that after remission or low disease activity had been achieved with TOF, dose reduction was associated with fewer flares than abrupt withdrawal, and reintroduction of the original regimen rapidly restored disease control in most flare cases (Mori and Ueki, 2019). The XANADU study further suggested that sustained remission after complete TOF withdrawal was achieved in only a minority of patients, whereas most patients who relapsed regained remission after resumption of TOF (Kubo et al., 2023b). An interesting finding was that patients with lower rheumatoid factor and anti-cyclic citrullinated peptide antibody levels before TOF discontinuation were less likely to flare, highlighting the potential importance of achieving low immunological disease activity before TOF withdrawal (Kubo et al., 2023b). In the previously mentioned KEIO-RA cohort, full-dose JAKi combined with MTX was associated with higher retention and fewer discontinuations due to lack of efficacy, without an apparent increase in safety issues, including across age groups (Takanashi et al., 2025).

Overall, these studies suggest that dose optimization in Japanese clinical practice should be individualized according to the specific clinical problem being addressed. Reduced dosing may improve tolerability in older, renally impaired patients, whereas full-dose therapy, particularly in combination with MTX, may be preferable when insufficient effectiveness is the primary concern.

## JAK selectivity and individualized within-class selection

JAK selectivity offers a useful pharmacologic framework, but current RA data do not support a simple translation of *in vitro* selectivity into a fixed clinical practice of efficacy or safety. In the previously mentioned FIRST registry, adjusted 2-year effectiveness did not differ meaningfully among TOF, BAR, UPA, and FIL, although UPA and FIL were more often selected as second-line agents in older patients with comorbidities (Sonomoto et al., 2026). Likewise, in a previous study of our department, JAK1-selective inhibitor showed better overall retention than pan-JAK inhibitor, but this difference appeared to be driven mainly by adverse-event-related discontinuation (Saito et al., 2024). The presence of baseline high disease activity, glucocorticoid use, and treatment with pan-JAK inhibitor was associated with reduced JAKi drug retention (Saito et al., 2024). Interestingly, in subgroup analyses restricted to JAK1-selective inhibitors, high baseline disease activity was not associated with lower drug retention, suggesting that JAK1-selective inhibitors may be less susceptible to retention loss in patients with highly active disease (Saito et al., 2024). Moreover, FIL may be associated with a numerically lower incidence of HZ than other JAKi (Table 1), making it a reasonable option in RA patients with a history of HZ, although this interpretation remains preliminary in the absence of direct head-to-head safety comparisons. Consistent with this concern, a large Japanese claims-based analysis by Takeuchi et al. showed numerically favorable persistence with FIL compared with other JAKi (Takeuchi et al., 2025). However, direct comparative safety analyses among individual JAKi remain inadequate and warrant further research.

Overall, the current evidence remains insufficient to support treatment selection based primarily on JAK selectivity. At present, it appears more appropriate to prioritize other patient-specific factors when choosing among JAKi.

## Comparison with non-Japanese real-world evidence

Although this review focuses on Japan-specific evidence, recent real-world data from other countries suggest that several observations from Japan are not entirely unique. In Italian multicenter studies, JAKi prescribing decreased after European Medicines Agency safety warnings, with a reduction in TOF prescriptions and a relative shift toward more selective JAKi, while real-world studies of UPA and TOF showed clinically meaningful effectiveness in routine care, including among patients with different cardiovascular risk profiles (Paroli et al., 2024; Lo et al., 2024; Priora et al., 2024). In the international JAK-pot collaboration, JAKi were not associated with a higher incidence of MACE than TNF inhibitors during the first 2 years of treatment, and overall real-world effectiveness was broadly comparable to that of TNF inhibitors in pooled registry analyses (Aymon et al., 2025; Lauper et al., 2022). Similarly, a nationwide French cohort study comparing JAKi with adalimumab provided reassuring data on MACE and VTE risks, including among patients at higher cardiovascular risk (Hoisnard et al., 2023). Data from the Hong Kong Biologics Registry further showed no increase in MACE or malignancy among JAKi users compared with TNF inhibitor users, although non-serious infections, including HZ, were more frequent (Mok et al., 2024). HZ appears to warrant particular attention in Asian populations, including Japanese patients, because pooled Asian analyses of JAKi-treated RA patients have reported higher HZ or HZ-related disease incidence than non-Asian reference populations (Yamanaka et al., 2016; Takeuchi et al., 2021b). In contrast, current evidence does not consistently suggest higher rates of MACE, VTE, or malignancy in Japanese or Asian patients.

Although Japanese studies directly linking standardized frailty indices to JAKi adoption or outcomes remain limited, recent non-Japanese real-world evidence suggests that frailty is increasingly recognized as a clinically relevant factor in the use of JAKi. In a recent administrative healthcare database study from Lombardy, Italy, patients initiating JAKi were older and had a higher electronic frailty index than TNF inhibitor users, suggesting that frailty may influence real-world JAKi adoption (Silvagni et al., 2025). In that study, JAKi exposure was not associated with significantly higher risks of cardiovascular events, MACE, or thromboembolic events compared with TNF inhibitors, whereas frailty itself was independently associated with these outcomes (Silvagni et al., 2025). In addition, frailty has been associated with an increased risk of serious infections among RA patients treated with bDMARDs or JAKi, (Singh et al., 2024). These findings support incorporating frailty assessment into baseline risk stratification, shared decision-making, and longitudinal monitoring when considering JAKi therapy, particularly in older or multimorbid patients.

Taken together, these non-Japanese real-world studies support the generalizability of a risk-optimized approach that considers not only drug class but also patient-level vulnerability, including frailty, comorbidities, cardiovascular risk, and susceptibility to infection. At the same time, Japanese evidence remains particularly informative given the older age structure, high representation of multimorbid patients, and the need for heightened caution for HZ in routine RA care.

## Conclusion

In conclusion, taken together with international trials and postmarketing analyses, the Japanese evidence base supports a risk-optimized approach to JAKi use in RA. Age is an important risk factor, but it should not be used in isolation. Thus, the comorbidity problem and other host-related vulnerabilities often appear more clinically informative than age alone. Across the class, HZ remains the most consistent safety signal, making vaccination and early monitoring practical components of routine practice. At the same time, currently available Japanese data suggest that older age alone does not necessarily disturb JAKi use, and that individualized dose adjustment may improve tolerability. Therefore, rather than seeking a single best JAKi, rheumatologists should individualize agent selection, dose, and monitoring according to patient-specific risk domains and therapeutic priorities, particularly in the increasingly older and multimorbid Japanese RA population.
