# Supplementary material for: Japanese evidence on Janus kinase inhibitors for rheumatoid arthritis: a narrative review of risk-optimized use
Source: Front Pharmacol. 2026 Jun 12;17:1842268. doi: 10.3389/fphar.2026.1842268 (PMC13299099; doi:10.3389/fphar.2026.1842268)
Supplement: Supplementary file 1 [file Supplementaryfile1.docx]

***Search strategie*s**

**Databases and time frame**

PubMed/MEDLINE and Ichushi-Web (Japan Medical Abstracts Society) were searched to identify Japan-specific evidence on Janus kinase inhibitors (JAKi) in rheumatoid arthritis (RA), including randomized trials, long-term extension studies, postmarketing surveillance studies, registry analyses, and observational studies.

The search was designed to capture evidence relevant to efficacy outcomes, treatment persistence, safety outcomes for current risk stratification, such as serious infections, herpes zoster, hospitalized infections, major adverse cardiovascular events, venous thromboembolism, malignancy, and laboratory abnormalities, as well as dose optimization, dose reduction, treatment sequencing, within-class JAKi selection, vaccination, comorbidity burden, renal impairment, glucocorticoid exposure, frailty-related vulnerability, and other patient-related risk factors in Japanese patients with RA.

Searches were conducted on 15 March 2026 for articles published from 1 January 2018 to 15 March 2026. Searches were limited to human studies. Articles in English or Japanese were considered.

**PubMed/MEDLINE search**

The following query was used with the publication date filter (2018/01/01:2026/03/05[dp]).

Search (PubMed):

("arthritis, rheumatoid"[MeSH Terms] OR "rheumatoid arthritis"[Title/Abstract]) AND ("Janus Kinase Inhibitors"[MeSH Terms] OR "janus kinase inhibitor*"[Title/Abstract] OR "jak inhibitor*"[Title/Abstract] OR "targeted synthetic dmard*"[Title/Abstract] OR "targeted synthetic disease modifying antirheumatic drug*"[Title/Abstract] OR "tsdmard*"[Title/Abstract] OR "tofacitinib"[Title/Abstract] OR "Xeljanz"[Title/Abstract] OR "baricitinib"[Title/Abstract] OR "Olumiant"[Title/Abstract] OR "upadacitinib"[Title/Abstract] OR "Rinvoq"[Title/Abstract] OR "filgotinib"[Title/Abstract] OR "Jyseleca"[Title/Abstract] OR "peficitinib"[Title/Abstract] OR "Smyraf"[Title/Abstract]) AND 2018/01/01:2026/03/15[Date - Publication] AND ("Japan"[MeSH Terms] OR "japan*"[Title/Abstract] OR "japan*"[Affiliation] OR "Japanese"[Title/Abstract] OR "Japanese"[Affiliation])

**Ichushi-Web search**

The following query was used with filters for human studies (CK=ヒト), publication years (DT=2018:2026), and original articles (PT=原著論文).

Search (Ichushi-Web):

(関節リウマチ/TH OR "rheumatoid arthritis"/AL) AND (JAK阻害薬/AL OR ヤヌスキナーゼ阻害薬/AL OR "Janus kinase inhibitor"/AL OR トファシチニブ/AL OR バリシチニブ/AL OR ウパダシチニブ/AL OR フィルゴチニブ/AL OR ペフィシチニブ/AL OR Xeljanz/AL OR Olumiant/AL OR Rinvoq/AL OR Jyseleca/AL OR Smyraf/AL OR tsDMARDs/AL OR tsDMARD/AL OR "targeted synthetic DMARD"/AL OR "targeted synthetic DMARDs"/AL OR 分子標的合成抗リウマチ薬/AL OR 分子標的合成DMARD/AL OR 標的合成DMARD/AL OR 標的合成抗リウマチ薬/AL) AND CK=ヒト AND DT=2018:2026 AND PT=原著論文
